# Supplementary material for: Direct comparison of spatial transcriptional heterogeneity across diverse Bacillus subtilis biofilm communities
Source: Nat Commun. 2023 Nov 20;14:7546. doi: 10.1038/s41467-023-43386-w (PMC10661151; doi:10.1038/s41467-023-43386-w)
Supplement: Supplementary file 1 — Supplementary information [file 41467_2023_43386_MOESM1_ESM.pdf]

Supplementary Information

Direct comparison of spatial transcriptional heterogeneity across diverse *Bacillus subtilis* biofilm communities

Yasmine Dergham[1,2], Dominique Le Coq[1,3], Pierre Nicolas[4], Elena Bidnenko[1], Sandra Dérozier[4], Maxime Deforet[5], Eugénie Huillet[1], Pilar Sanchez-Vizuete[1], Julien Deschamps[1], Kassem Hamze[2*], Romain Briandet[1*]

[1]Université Paris-Saclay, INRAE, AgroParisTech, Micalis Institute, 78350 Jouy-en-Josas, France.
[2]Lebanese University, Faculty of Science, 1003 Beirut, Lebanon.
[3]Université Paris-Saclay, Centre National de la Recherche Scientifique (CNRS), INRAE, AgroParisTech, Micalis Institute, 78350 Jouy-en-Josas, France.
[4]Université Paris-Saclay, INRAE, MAIAGE, 78350 Jouy-en-Josas, France.
[5]Sorbonne Université, CNRS, Institut de Biologie Paris-Seine, Laboratoire Jean Perrin, Paris, France.
*corresponding authors: romain.briandet@inrae.fr (R.B.), kassem.hamze@ul.edu.lb (K.H.).

1. **Supplementary Figures**
2. **Supplementary Notes**
3. **Supplementary Table**

1- Supplementary Figures

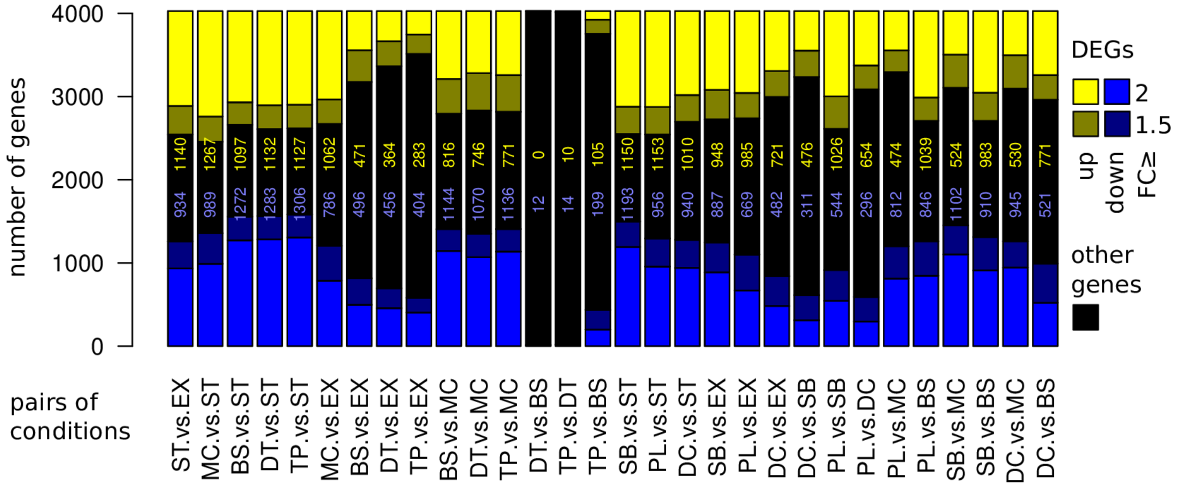

Figure 1: Comparison of differentially expressed genes (DEGs) between the different *B. subtilis* populations in the RNAseq analysis. Out of 4028 genes, the number of DEGs in pairwise comparison of different *B. subtilis* populations (q-value≤0.05, | log2FC≥1) are reported in yellow (upregulated) and in blue (downregulated). Supplementary Data 1 contains the comprehensive compilation of statistically significant correlations involving upregulated and downregulated genes across various populations.

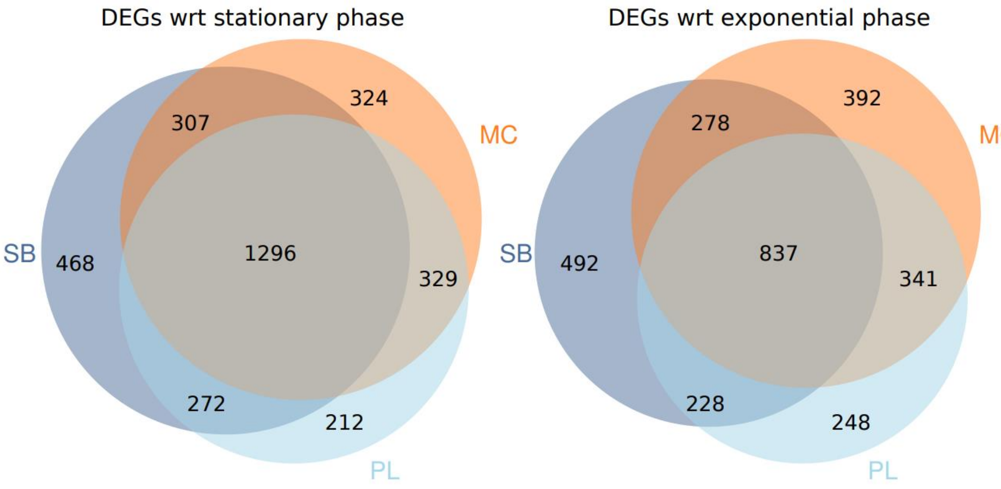

Figure 2: Venn diagram comparing the sets of differentially expressed genes (DEGs) in the different biofilm populations. The left and right diagrams represent respectively the relationships between sets of DEGs with respect to the stationary and exponential phase conditions. DEGs are defined using cut-offs q-value≤0.05 and log2FC≥1 (up-regulation) or log2FC≤-1 (down-regulation). A gene is represented as common between two biofilm

populations (*i.e.* belonging to the intersection set) if it is either up- or downregulated compared to the reference (stationary or exponential phase).

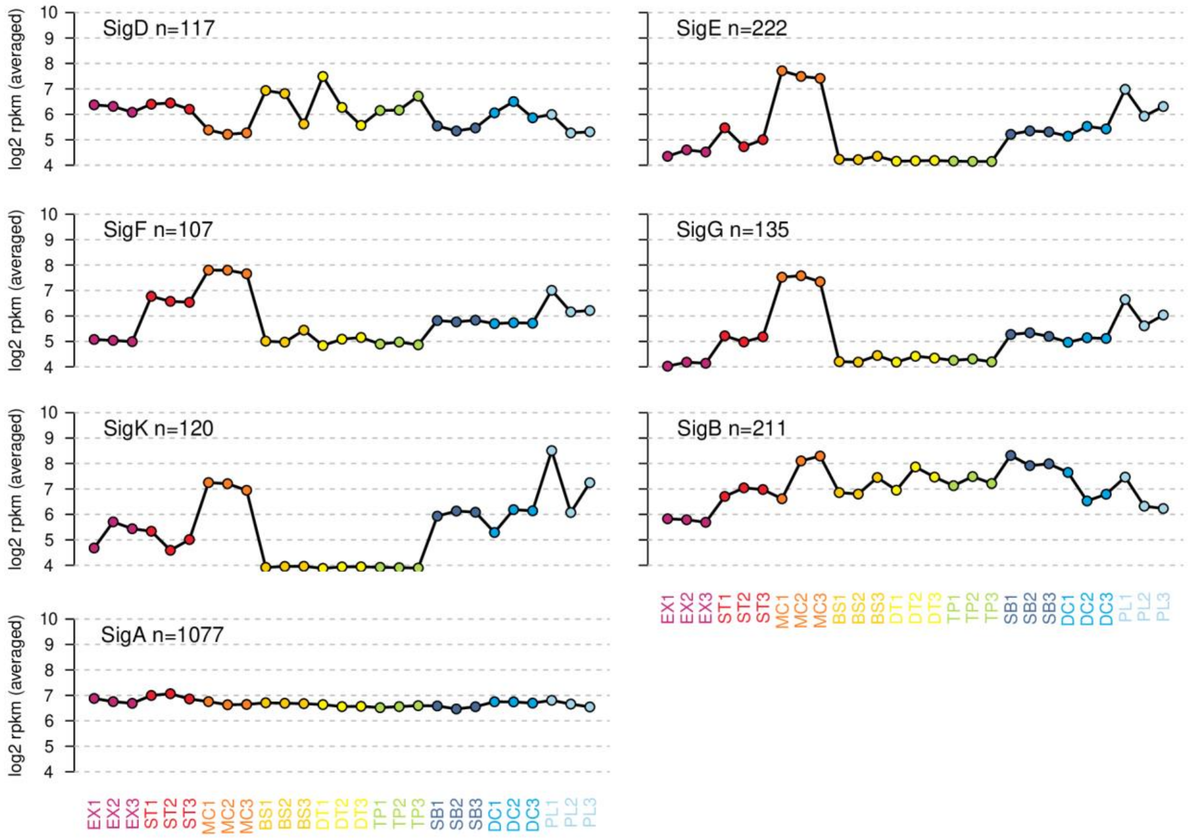

Figure 3: Expression profiles of Sigma factor regulons. Each subplot represents the profile across all samples of a particular Sigma factor. Profiles are obtained as the log2(rpkm+5) averaged over genes listed in the *Subti*Wiki database as belonging to the corresponding specific regulon, consistent with the expression profile of the Sigma factor itself (Supplementary Data 1). The number of genes ranges from 107 for SigF to 1077 for SigA (as reported in each subplot).

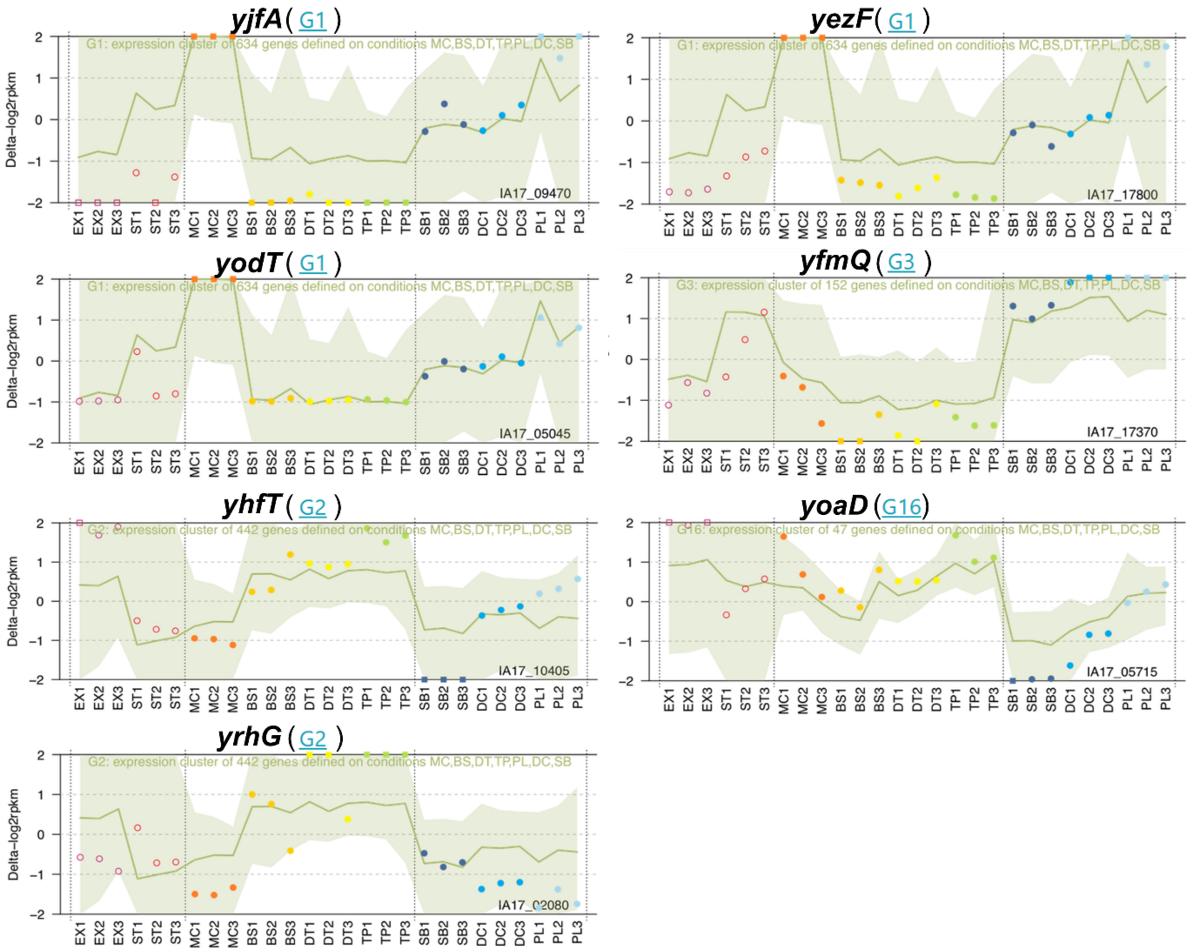

Figure 4: Investigating the intriguing expression profiles of some *B. subtilis* genes with unknown functions. The following graphs were extracted from the interactive online website Genoscapist [https://genoscapist.migale.inrae.fr/seb_bsubbiofilm/].

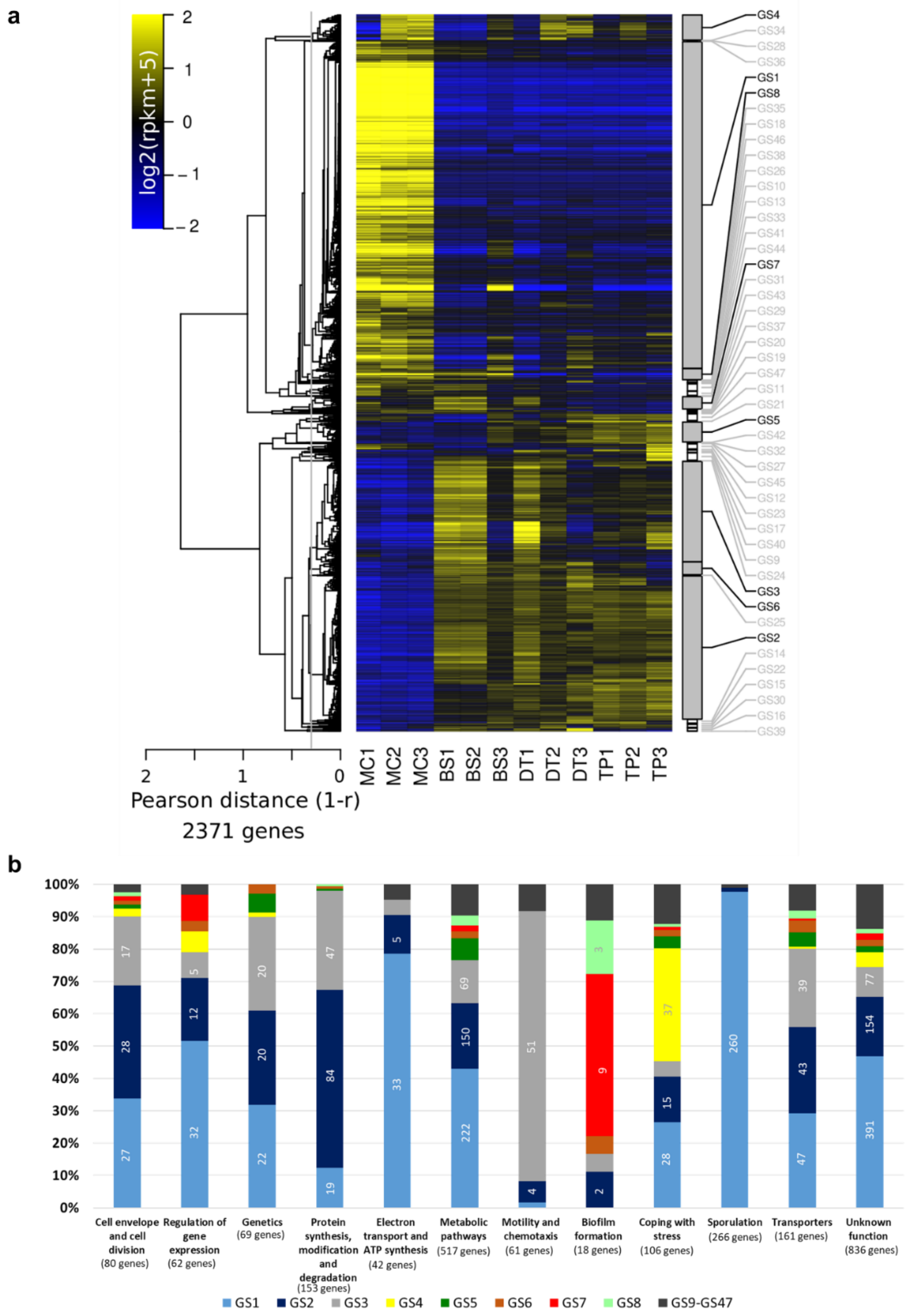

Figure 5: Transcriptome remodelling of differentially expressed genes during swarming. (a) Heatmap representation of the relative variation in expression level across samples for DEGs (2371 out of 4028) identified in the 6 pairwise comparisons of 4 distinct localisations: mother colony (MC), base (BS), dendrites (DT), and tips (TP). The colour code reflects the comparison with the mean calculated for each gene across the 12 samples (log2 ratio). The hierarchical clustering tree shown on the left side of the heatmap (average link) was clipped at an average Pearson correlation of 0.7 (vertical grey line) to define the expression clusters shown on the right side of the heatmap. The clusters are named by decreasing size (from GS1 to GS47) and those containing more than 30 genes are highlighted (name in black) (Supplementary Data 1). **(b)** Distribution in expression clusters for genes in functional categories derived from *Subti*Wiki.

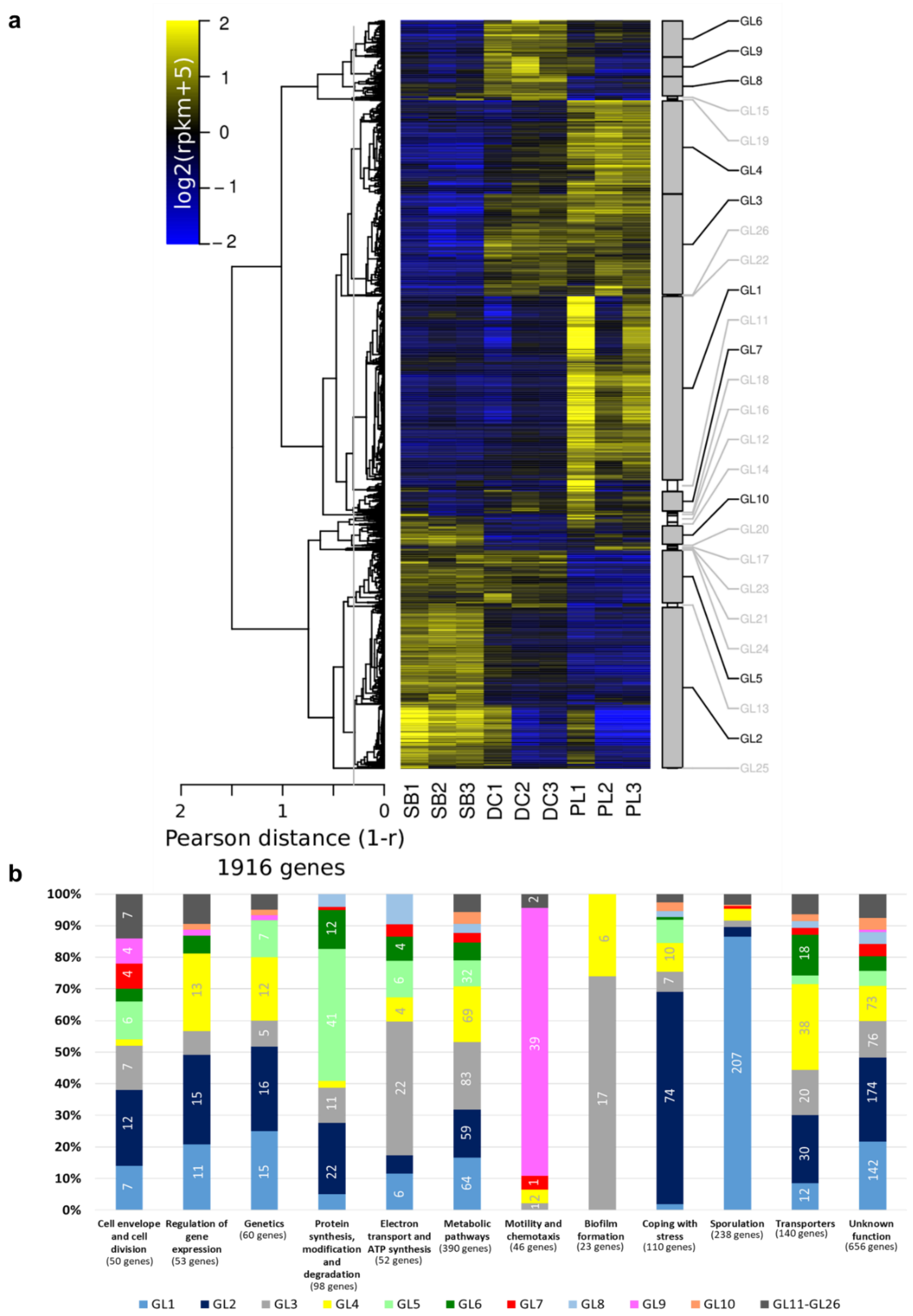

Figure 6: Transcriptome remodelling shows that half of the genome is differentially expressed between the floating pellicle and the submerged biofilms coexisting in the same microplate well. (a) Heatmap representation of the relative variations of expression level across samples for DEGs (1916) identified in the three pairwise comparisons of three different localisations (SB, DC, PL). Clusters were named (from GL1 to GL26) by decreasing sizes and those containing more than 30 genes are highlighted (Supplementary Data 1). **(b)** Distribution into expression clusters for genes in *Subti*wiki-derived functional categories.

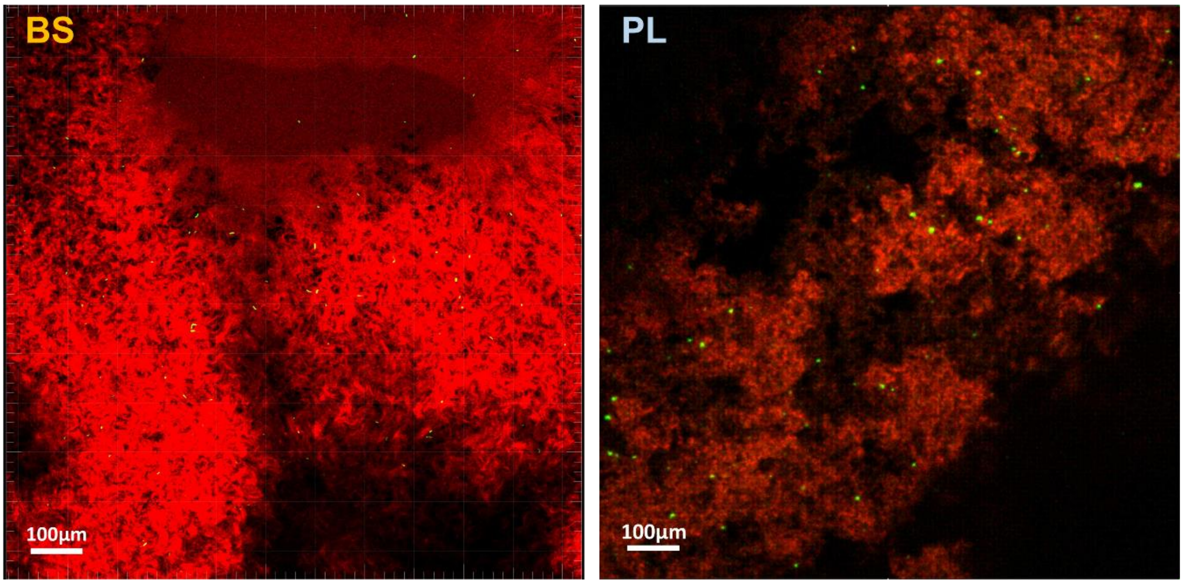

Figure 7: *In situ* spatial monitoring reveals the transition from glycolytic to gluconeogenic regime at the cellular level. Spatial confocal imaging using strain GM3900 reporting transcription of *cggR-gapA* by mCherry (in red) and of *gapB* by Gfp (in green), for base (BS) and pellicle (PL), from the swarming and static liquid models, respectively, after 24 hours of incubation at 30°C. The figure depicts cells in a gluconeogenic regime (green spots) within a population primarily under glycolytic regime (red). The same protocol as for the transcriptome analysis was used, except for the use of 96-well instead of the 12-well microplates for the static liquid model. Three replicate observations were performed independently for each model.

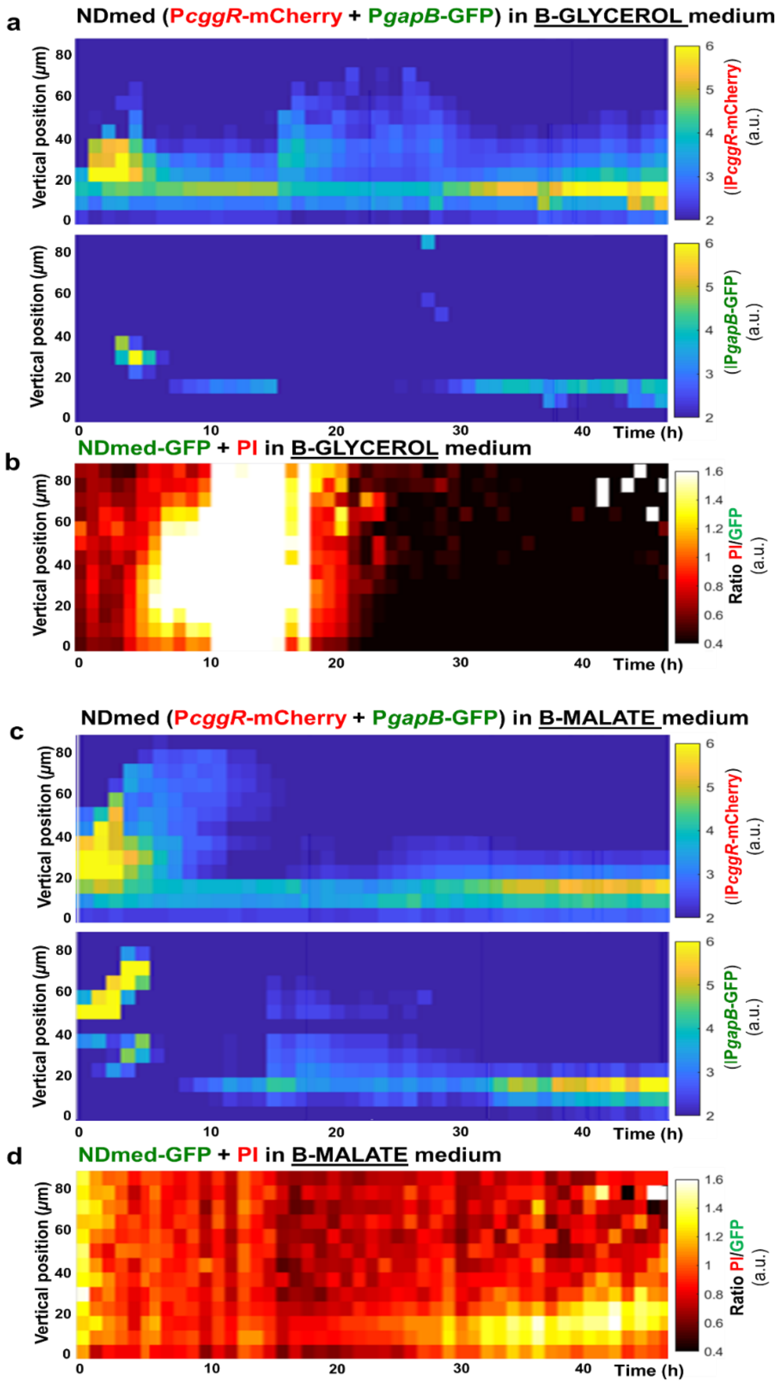

Figure 8: A correlation between carbon metabolism and dead cells. (a, c) Strain GM3900, reporting the transcription of *cggR* (*gapA*) with mCherry (in red) and of *gapB* with Gfp (in green), was cultivated in synthetic B-medium. Glucose was replaced with either glycerol (0.5% final concentration) or malate (0.5% final concentration). Kymographs depict the expression intensity of transcriptional reporter fusions for *cggR* and *gapB* genes along a spatiotemporal scale, colour-coded for representation. **(b, d)** Using NDmed-GFP (GM3649) and propidium iodide (PI) for permeable cell staining, the same protocol as used for transcriptome analysis was applied, except employing 96-well microplates instead of 12-well plates, and substituting glucose with glycerol or malate. A multidimensional kymograph illustrates dead cell intensity, obtained from the dead/living cell ratio, based on spatial localization and time. Kymographs are representative of three replicates.

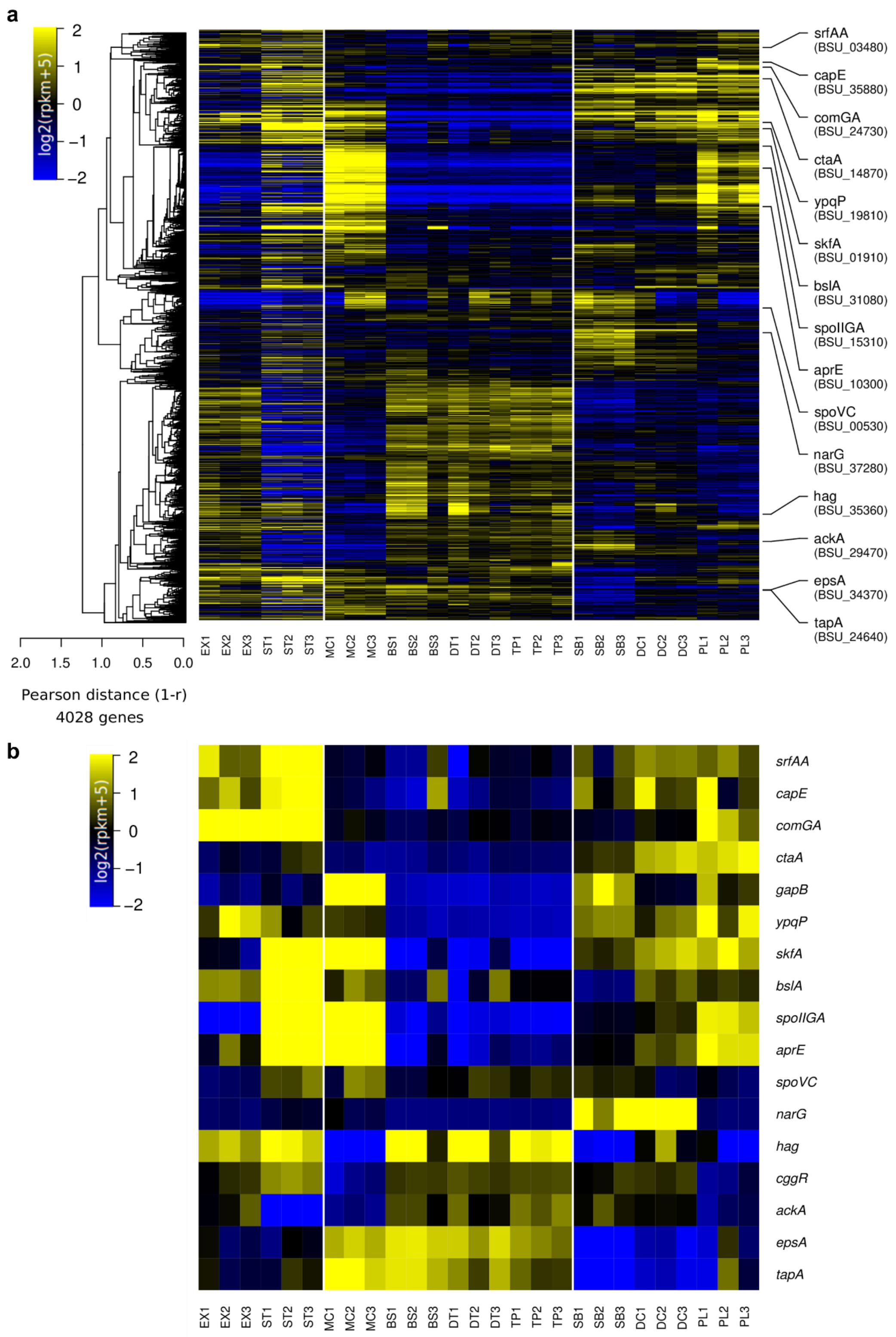

Figure 9: Global heatmap for all the different populations, pinpointing selected genes for reporting. (a) Global heatmap representation for the 4028 genes within NDmed in the spatially selected surface-associated populations. The colour code reflects the gene-to-average (log2 ratio) against the average calculated across all conditions, excluding the planktonic populations. On the far right are the selected and transcriptionally reported genes, representing different physiological activities potentially present in a biofilm. **(b)** A heatmap representation for the selected genes.

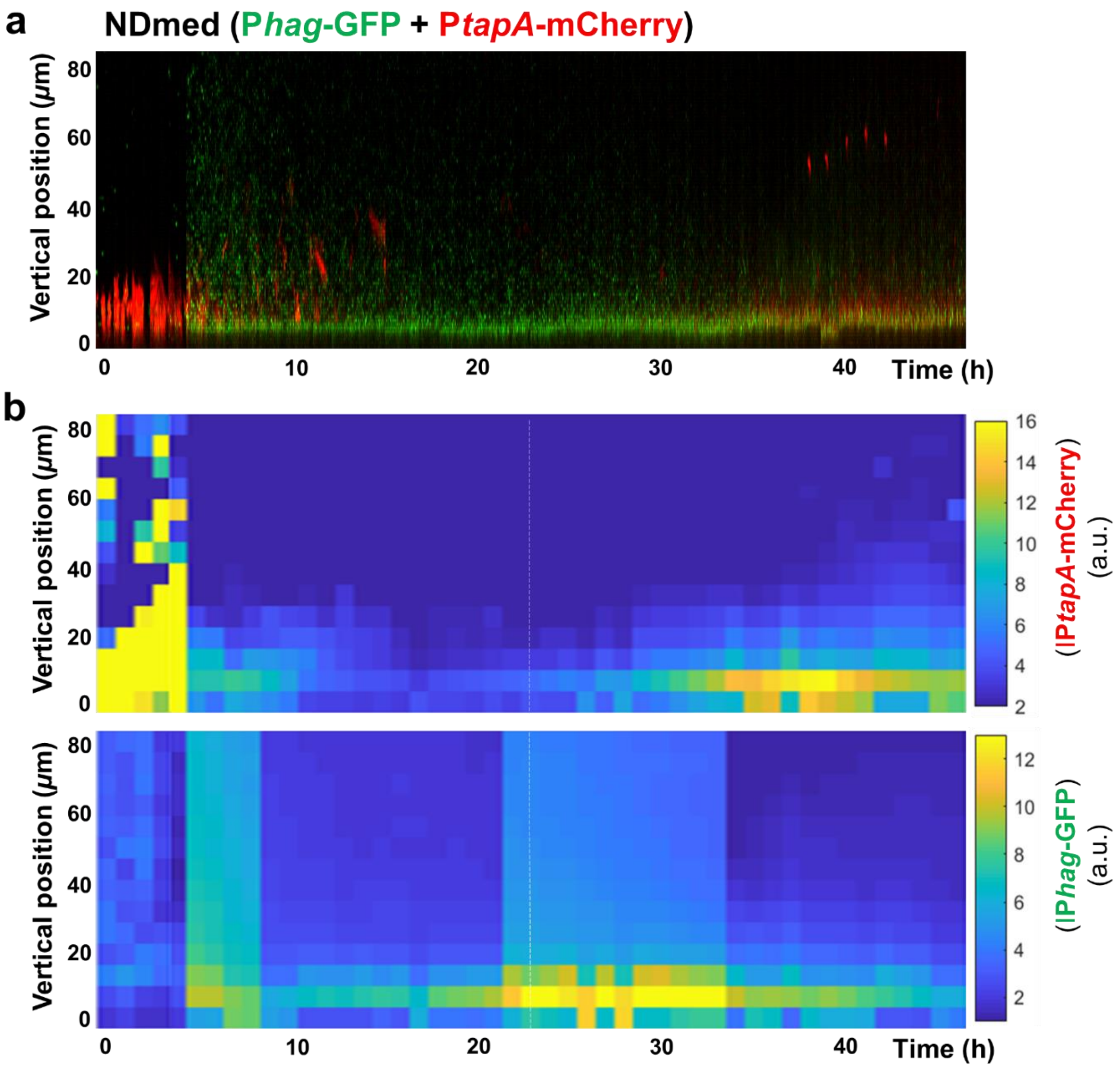

Figure 10: Spatiotemporal imaging for the submerged biofilm compartment. (a) Sections from real-time confocal imaging (x 20µm, y 50µm, z 80µm) for 48 hours, image every one hour, using strain GM3924 reporting transcription of *tapA* by mCherry (in red) and of *hag* by Gfp (in green), with the same protocol used for the transcriptome analysis, except the usage of 96-well microplates instead of the 12-well (Supplementary Movie 6). **(b)** Kymographs representing by a colour code the intensity of the expression for the transcriptional reporter fusions to the *tapA* and *hag* genes along a spatiotemporal scale. The white dotted line in each kymograph represents the time (24 hours) corresponding to the RNA-seq analysis. Three biological replicates were performed.

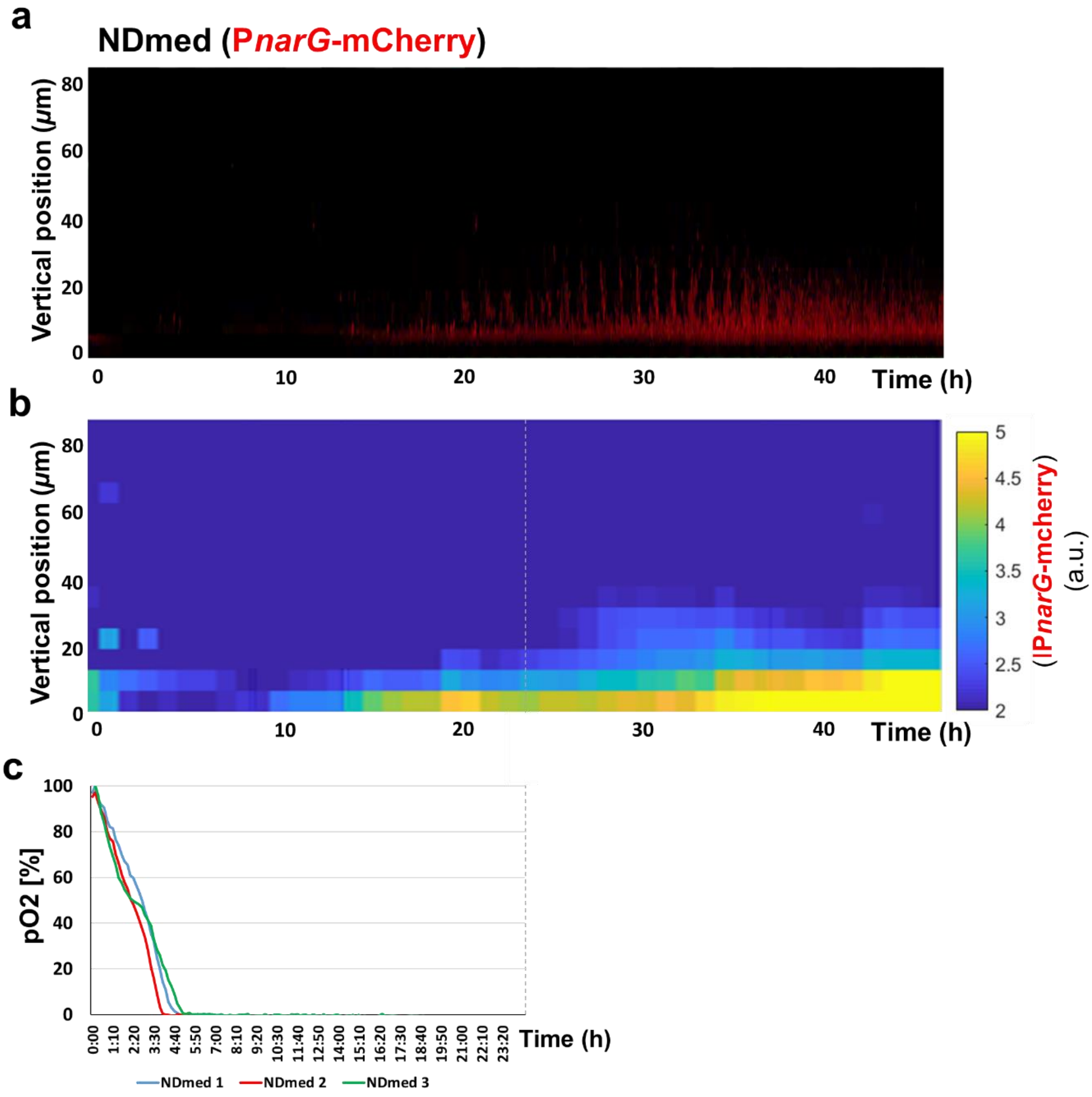

Figure 11: Spatiotemporal imaging for the submerged biofilm compartment. (a) Sections from real-time confocal imaging (x 50µm, y 50µm, z 80µm) for 48 hours, image every hour, using strain GM3907 reporting transcription of the *narG-I* operon by mCherry (in red), with the same protocol used for the transcriptome analysis, except the usage of 96-well microplates instead of the 12-well. **(b)** Kymographs representing by a colour code the intensity of the expression for the transcriptional reporter fusion to the *narG-I* operon promoter along a spatiotemporal scale. The white dotted line in each kymograph represents the time (24 hours) corresponding to the RNA-seq analysis. Three biological replicates were performed (Supplementary Fig. 14). **(c)** Using an OxoPlate OP96F (PreSen, Regensburg, Germany) the amount of oxygen was followed over time during the submerged biofilm development. The oxygen calculation was similar to that in B. Hutter and G. T. John, *Curr Microbiol*, 2003, https://doi.org/10.1007/s00284-003-4095-4. The three curves represent the biological replicates. Source data are provided as a Source Data file.

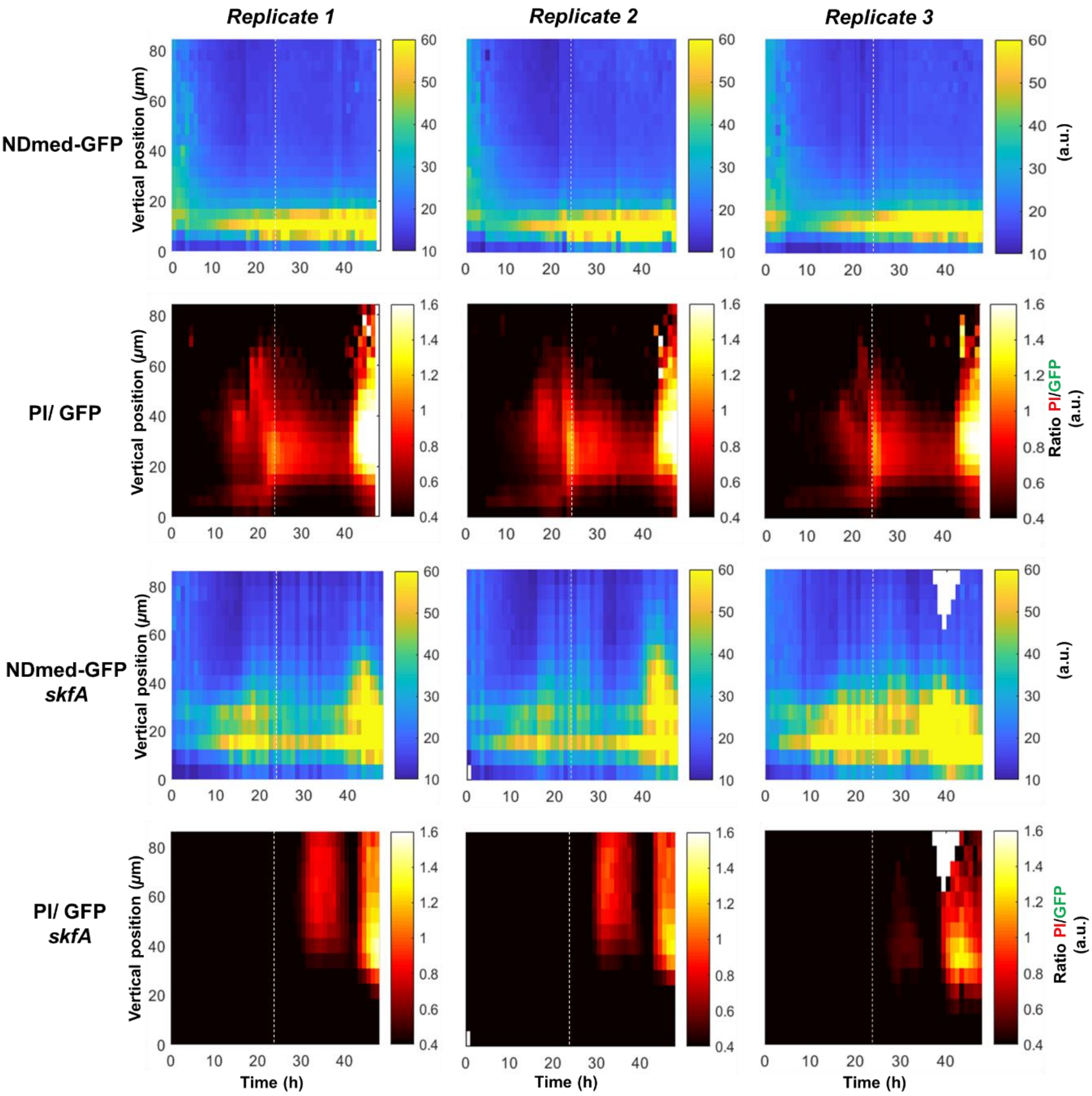

Figure 12: Comparative kymographs illustrating spatial and temporal dynamics of live (GFP) and dead (PI) subpopulations of *B. subtilis* NDmed-GFP and derivative *skfA* mutant. This figure depicts the lack of the initial death wave within a submerged biofilm monitoring setup involving a *skfA* mutant context. Nevertheless, the second wave of death did manifest. The kymographs are derived from three replicates, each extracted from distinct biological replicate movies. The white dotted line in each kymograph represents the time (24 hours) corresponding to the RNA-seq analysis. The *skfA::kan*^{R} mutation was introduced into the NDmed-GFP strain by transformation with chromosomal DNA from strain BBK01910 (Koo BM et al., *Cell Systems*, 2017, https://doi.org/10.1016/j.cels.2016.12.013).

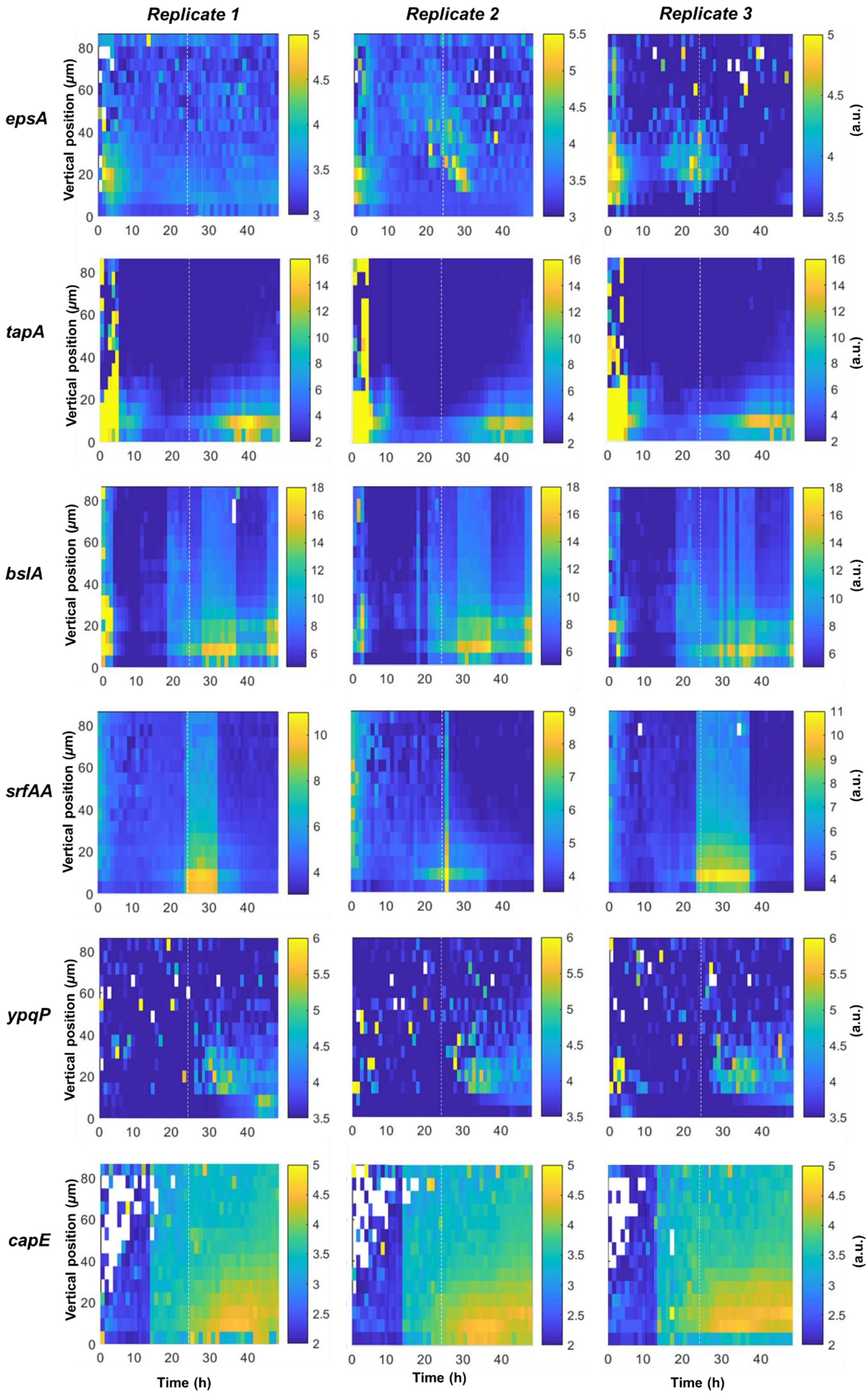

Replicate 1
Replicate 2
Replicate 3
epsA
tapA
bslA
srfAA
ypqP
capE
Vertical position (μm)
Time (h)
(a.u.)

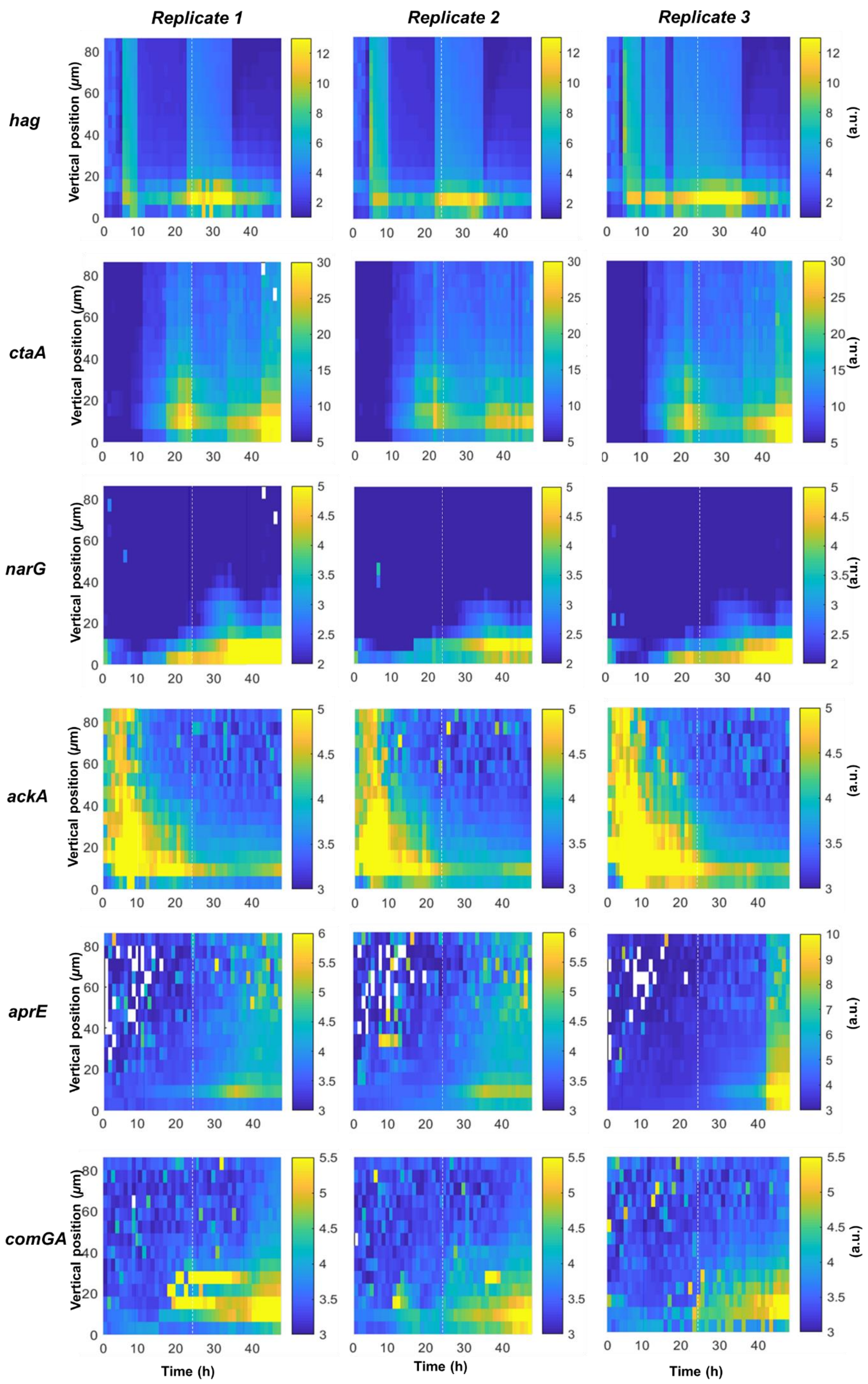
Replicate 1
Replicate 2
Replicate 3
hag
ctaA
narG
ackA
aprE
comGA
Vertical position (μm)
Time (h)
(a.u.)

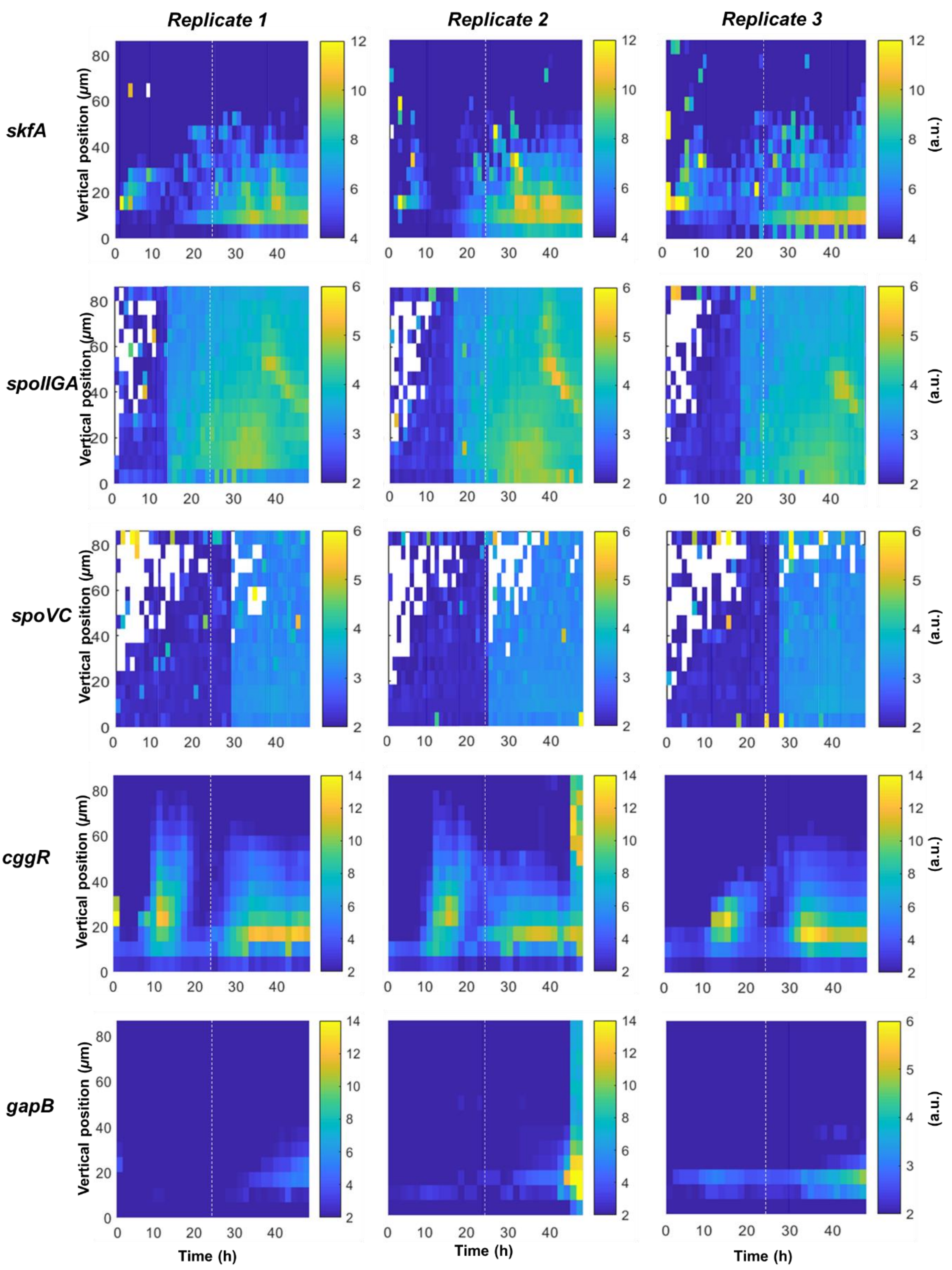

Figure 13: Multigene space-time transcriptional analysis in submerged biofilms. Three replicates of space-time kymographs of fluorescent reporters for transcription of 17 genes/operons in submerged biofilm. The white dotted line in each kymograph represents the time (24 hours) corresponding to the RNA-seq analysis. The full raw image dataset used in BiofilmQ for their generation is available at: https://doi.org/10.57745/Z511A6

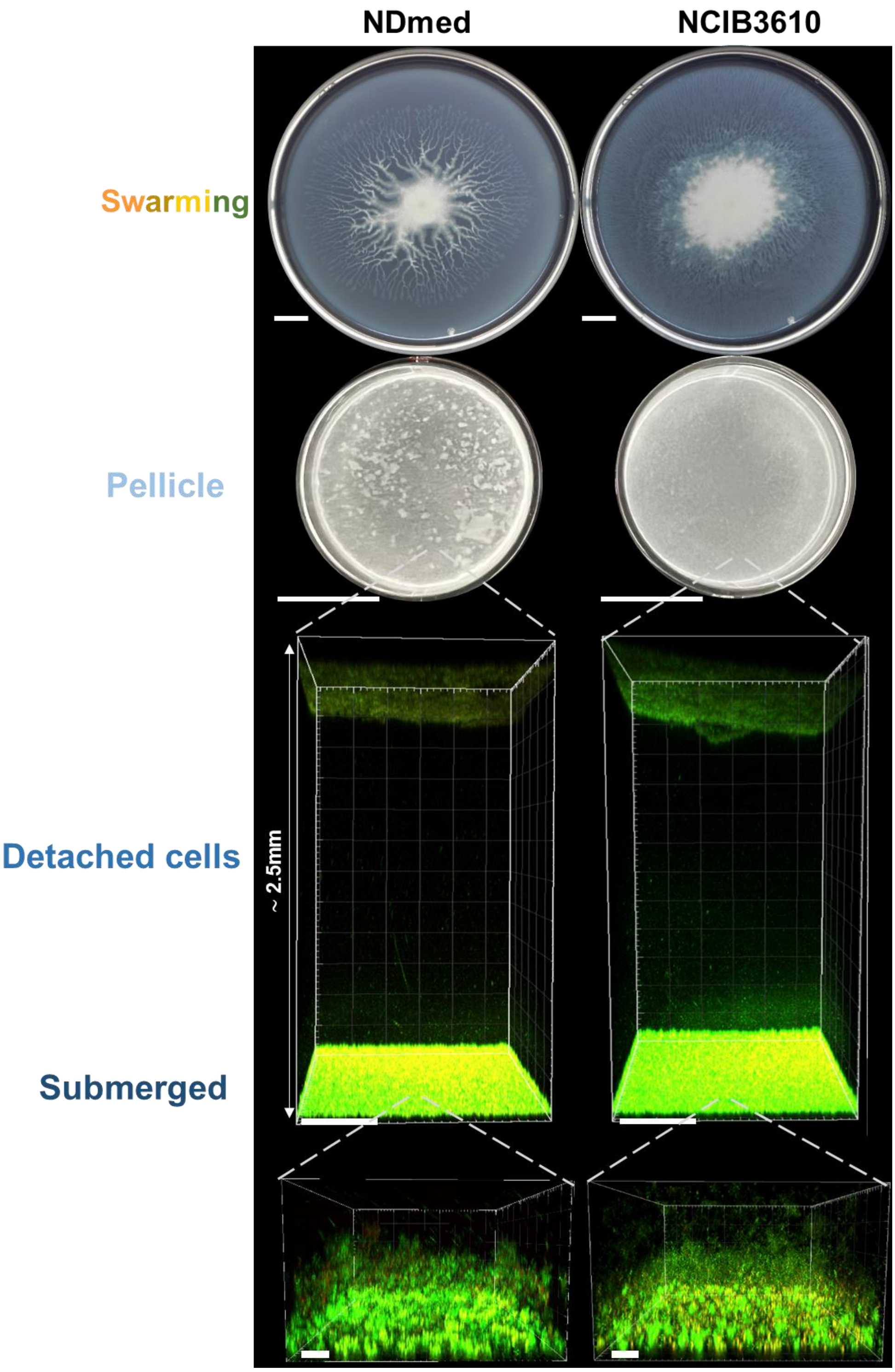

Figure 14: Comparative analysis of two *B. subtilis* strains using a multi-culture approach. This figure presents a comparative study of two distinct *B. subtilis* strains, employing a multi-culture experimental framework. Both strains, NDmed and NCIB3610, were cultivated under uniform conditions using the synthetic B-medium, as detailed in the materials and methods section. For confocal imaging, Syto9 (live cells, in green) and PI (dead cells, in red) staining were used. Scale bars indicate 1 cm for colonies and pellicles, 400 µm for submerged, and 20 µm for the zoomed submerged figure.

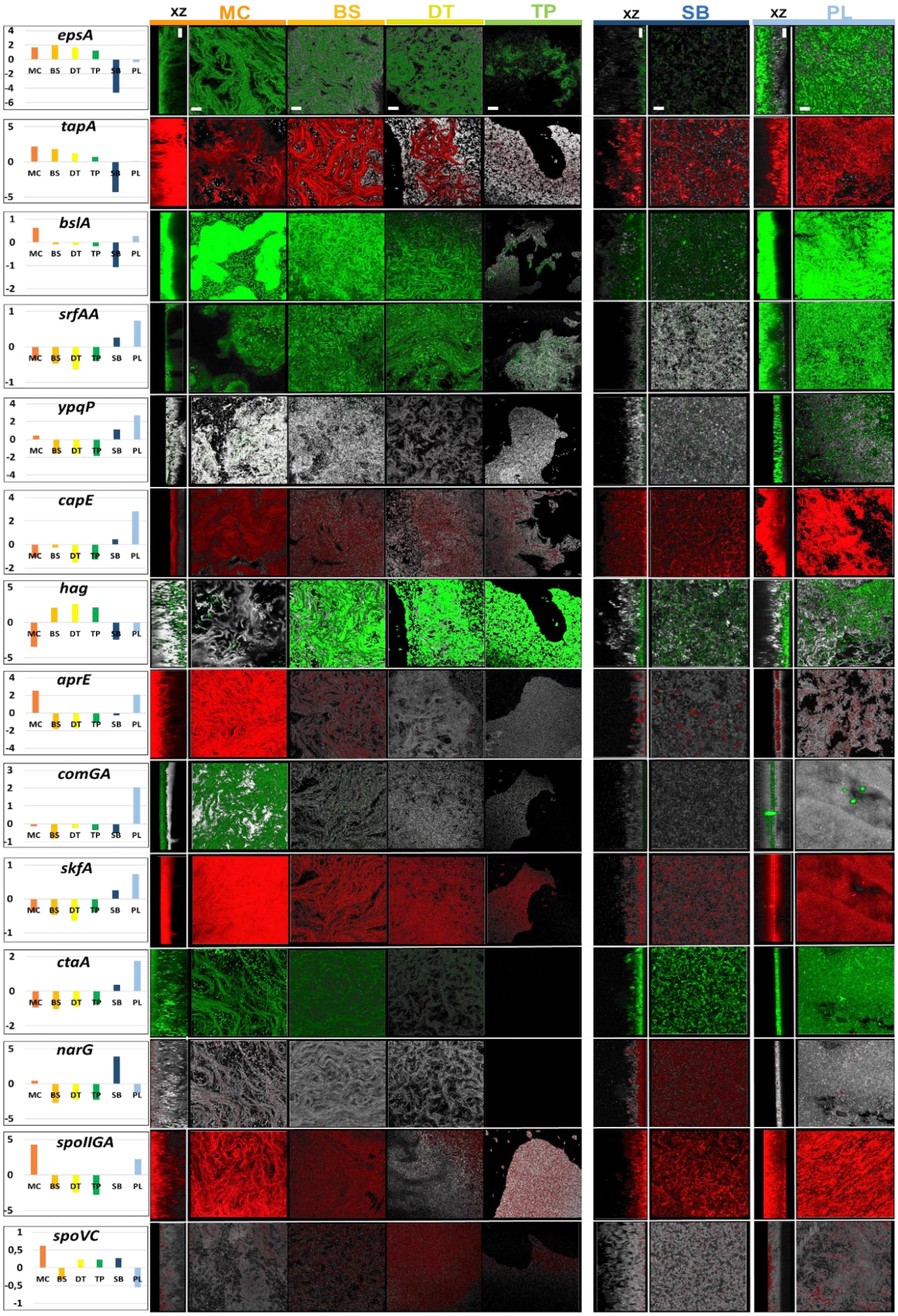

Figure 15: From mesoscopic to microscopic scale. The RNAseq data is represented on the most left panels with a 1og2FC scale of an average of the three biological replicates compared

to the average of all presented conditions together. The transcriptome results of *epsA*, *capE* and *narG* are used as representatives for their corresponding operon *epsA-O*, *capB-E* and *narG-I*, respectively. The expression of the gene is visualised using confocal imaging, with Gfp fluorescence indicated in green and mCherry fluorescence shown in red. The grey contrast was achieved either through chemical staining (using Syto9 or Syto61) or by expressing a second reporter gene fusion in strains containing such a fusion. For swarming plates (MC, Mother Colony; BS, Base; DT, Dendrites; TP, Tips) and static liquid cultures (SB, Submerged; PL, Pellicle), images represent a section (x and y 50µm and z 30µm); the scale bar represents 20µm. For the biofilm models (MC, SB and PL), a projection through the xz plane is presented on the left of each image; the vertical scale bars indicate the bottom level of the surface associated communities, in contact with the agar surface, solid surface or liquid surface, respectively. Images are representatives of the major phenotype from at least three replicates for each population.

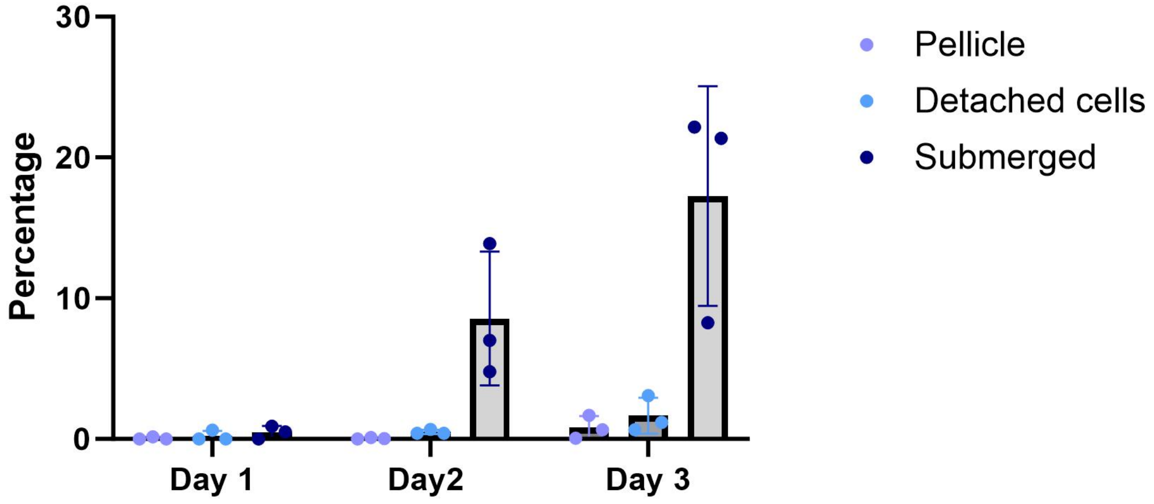

Figure 16: Counting spores in various populations within a static liquid model. Samples were collected from these diverse populations and were either subjected to heat treatment (80°C for 10 minutes) or left untreated. These samples were plated on a solid rich medium. Counting the resulting colonies allowed to estimate the percentage of heat-resistant spores within each population (p-value < 0.05). Each test was performed in triplicate. Source data are provided as a Source Data file.

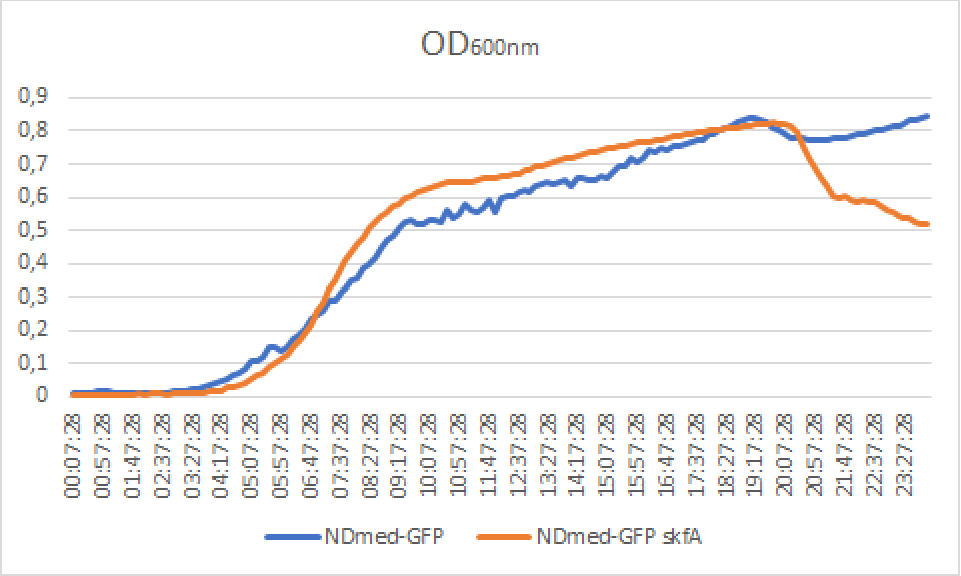

Figure 17: Differential growth curves of NDmed-GFP and a *skfA*-mutant derivative in planktonic culture. Strains were cultured in B-medium at 37°C with continuous shaking in a 96-well plate. Optical density (OD_{600nm}) readings were taken using a Biotek reader. Source data are provided as a Source Data file.

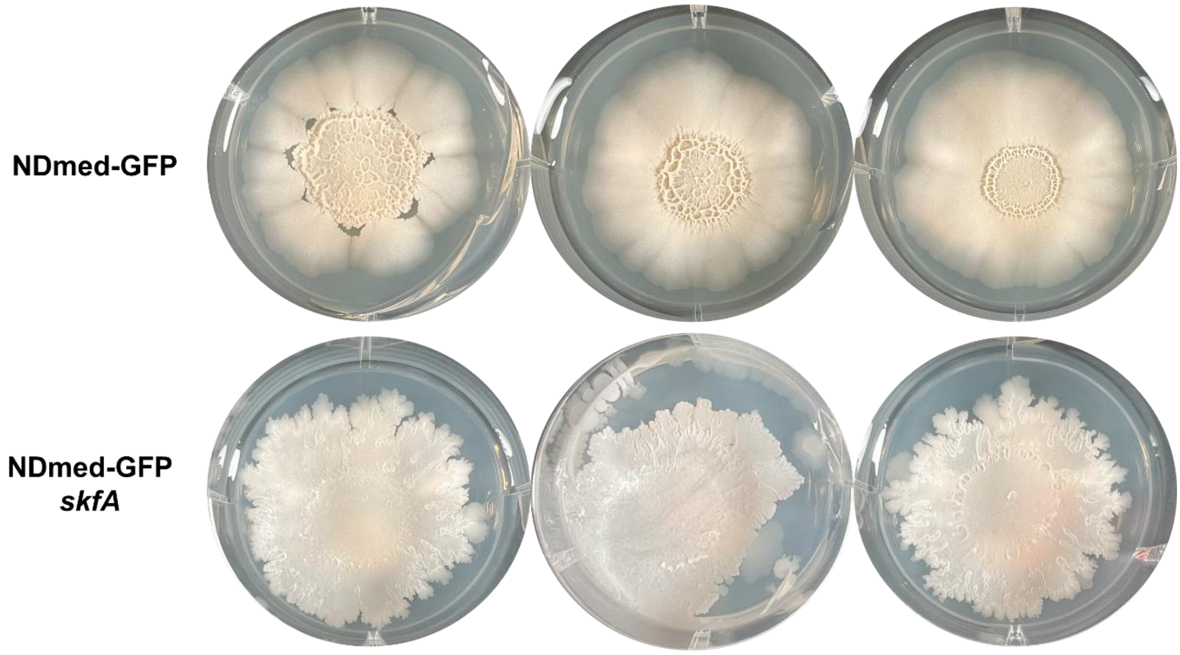

Figure 18: Colony comparison between *B. subtilis* NDmed-GFP and *skfA* mutant. The figure depicts three biological replicates of strains cultured on 1.5% agar synthetic B-medium at 30°C for 4 days.

2- Supplementary Notes

<u>Supplementary Note 1: Analysis of the swarming model RNAseq data</u>

A retrospective view on the adjacent spatial compartments of a swarm shows differential gene expression

Transcriptome analysis of four different localisations (MC, BS, DT, and TP) reflecting the successive stages of swarming revealed distinct patterns of gene expression in the corresponding cell populations. From the 4028 genes of the *B. subtilis* NDmed, 2371 genes are differentially expressed between the four localised compartments of a swarm and grouped according to the similarity of their gene expression, represented as a heatmap in Supplementary Fig. 5a. There are 47 groups of genes differentially expressed within the swarming model (GS1 to GS47). Supplementary Fig. 5b, represents a functional category percentage of the 2371 genes across the different conditions of a swarm.
Most of the sporulation genes (507 out of 629 genes from the *Subti*Wiki lifestyle category "Sporulation") were grouped in the largest GS1 cluster (1082 genes). This cluster includes genes involved in sporulation regulatory circuits and those required for different stages of spore formation. Comparative analysis of the gene expression patterns indicates an effective activation of the sporulation process in the mother colony population compared to the other three.
A gene expression "signature" characteristic of mother colony, induced either by early or late sporulation-specific sigma factors SigE (190 of 221), SigF (84 of 107), SigG (116 of 135) and SigK (103 of 120) as well as specific TFs (*GerE*, *SpoVT*, *SpoIIID*), indicates a high heterogeneity of the mother colony population with respect to the sporulation process.

Example:

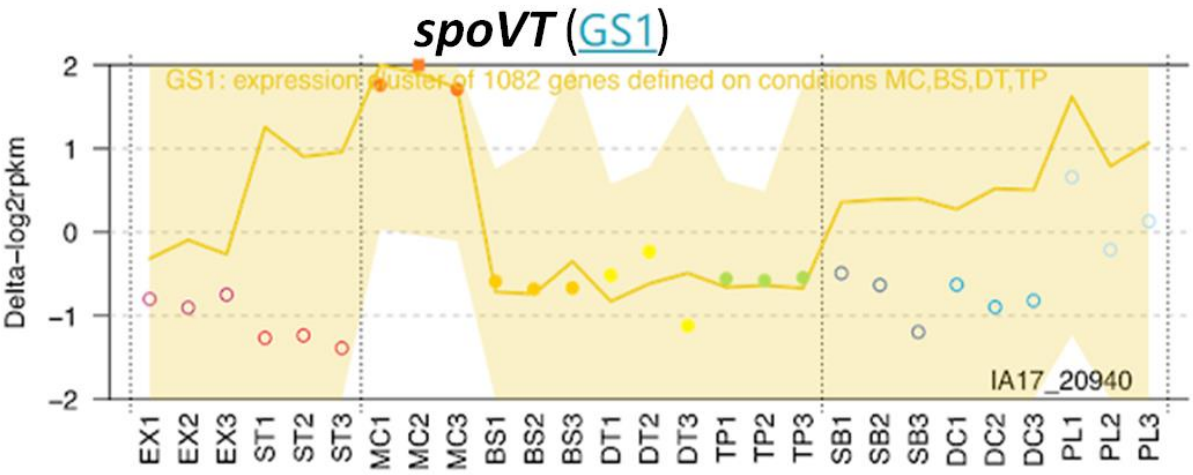

Expression profile across the different populations of spoVT (transcription regulator of sigG-dependent genes). The graph was extracted from Genoscapist.

In line with this, genes from the SigD regulon (73 out of 117), which encode proteins that ensure the mutually exclusive program of motility and chemotaxis and are partially repressed by the master regulator of sporulation Spo0A (37 out of 79), showed opposite behaviour, as being downregulated in the mother colony population compared to swarmer populations, and were grouped into the GS3 cluster.

Example:

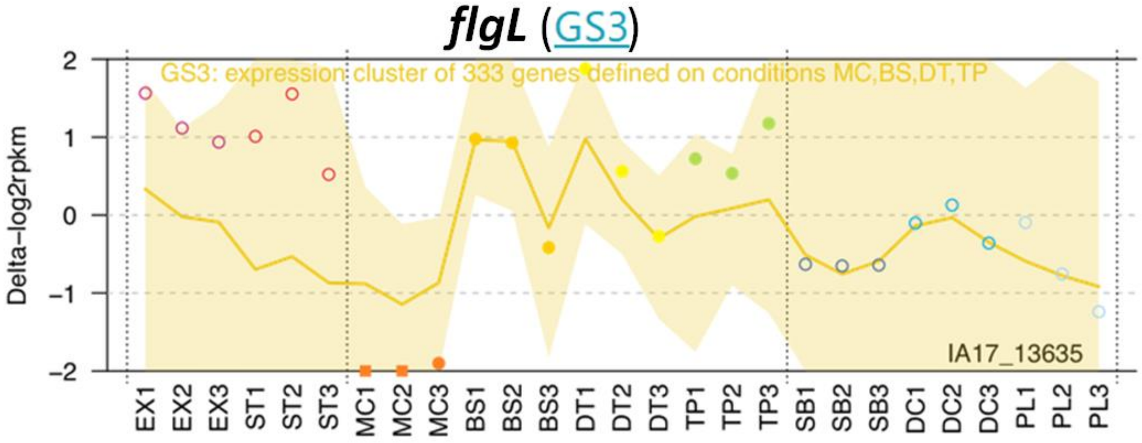

Expression profile across the different populations of flgL (flagellar hook-filament junction protein). The graph was extracted from Genoscapist.

The second group of genes overrepresented in the GS1 cluster and characterised by increased expression levels in the mother colony population includes genes involved in the utilisation of specific carbon sources (109 out of 243). These genes are known to be repressed by the carbon catabolite control protein CcpA.

Example:

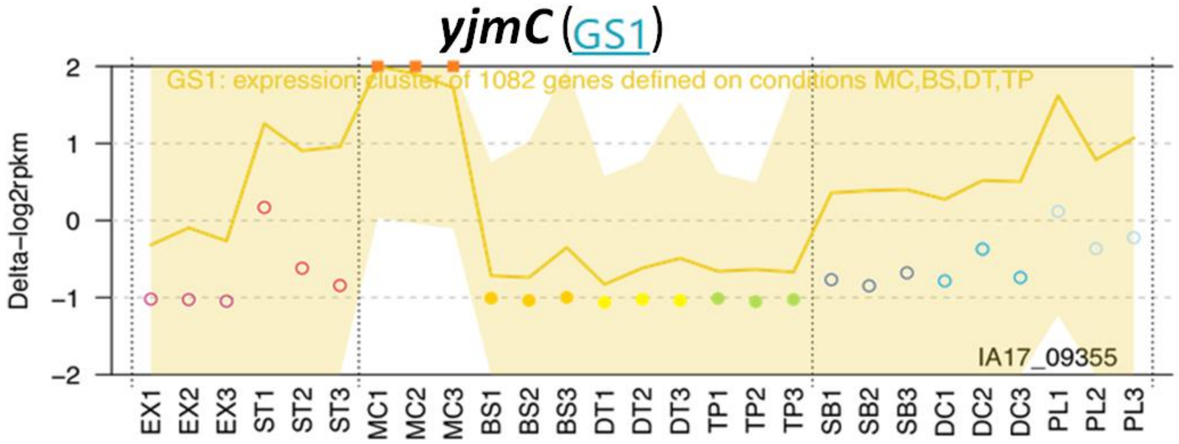

Expression profile across the different populations of the yjmC-uxaA operon (essential for galacturonate utilisation). The graph was extracted from Genoscapist.

A similar behaviour (strong upregulation in the MC population compared to other swarming populations) was found for the PhoP gene regulon (40 out of 58 genes). The PhoP-activated genes were grouped into the GS1 cluster.

The majority of genes (98 out of 140) known to be negatively regulated by the second stress adaptation mechanism, the stringent response, were downregulated in the mother colony population and grouped into the GS2 (56 out of 140) and GS3 (42 out of 140) clusters. In addition to genes belonging to the category "protein synthesis, modification and degradation" (e.g., ribosomal proteins, translation factors), the GS3 cluster includes genes belonging to the category "information processing" (e.g., *rpoA*, *nusA*). Accordingly, genes (16 out of 24) from the purine biosynthesis pathway controlled by the PurR repressor, whose binding activity is known to be stimulated by the stringent response, were downregulated in the mother colony population compared to the three. In addition, the GS2 cluster contains 34 out of 86 tRNA genes that were downregulated in MC.

Two groups of genes encoding the main components of the biofilm matrix: the *epsF-G-H-I-K-L-N-O* genes and the *tapA-tasA* operon showed slightly different expression patterns with a common feature of different levels of downregulation in the tips population compared to the other three, so they were grouped into clusters GS7 and GS8, respectively.

Similarly, genes from the category "Biosynthesis of antibacterial compounds" category (11 out of 53), which are involved in the production of the antibacterial compounds plipastatin, bacillaene and subtilosin were downregulated in the tips population and grouped in the GS8 cluster.

Comparison between adjacent compartments of a swarm highlights some genes that are differentially expressed (Supplementary Fig. 1). Between the base and the mother colony (BS vs MC) there are 815 genes upregulated and 1140 genes downregulated, most of which have been described in the GS1. In the dendrites, 12 genes are downregulated compared to the base (DT vs BS), *ppsD-E*, *rpsNB*, *yxeG*, *yrpE*, *folEB-yciB* and the *znuACB* and *pftAB* operons. In addition, 14 genes are downregulated (*veg*, *ykoY*, *ydzJ, spoVS, yydF, sspF, yqzM, tRNA-Met, tRNA-Thr, tRNA- Ser, tRNA-Asp, tRNA-Val, and 2 genes tRNA-Arg*) and 10 upregulated (*tRNA-His, ytbE, ytbD, rbsD, melE, melR, ldh, cydA, ydgH and ydgG*) in the tips when compared to the dendrites (TP vs. DT). The *ycdA* gene, encoding a lipoprotein required for swarming motility, shows gradual upregulation by a Log2FC from one localised compartment to its adjacent one going from the mother colony to the tips.

Supplementary Note 2: Analysis of the static liquid model RNAseq data

Half of the genome is differentially expressed between the floating pellicle and the submerged biofilms coexisting in the same microplate well

In this study, we have separately collected the submerged (SB), the pellicle (PL) as well as the detached cells (DC), a compartment between the SB and PL. From the 4028 genes, 1916 are differentially expressed between the three localised compartments clustered by 26 groups (GL1 to GL26) with functional category percentage, represented in Figure S4.

Groups GL6, GL9, and GL8 (Supplementary Fig. 4a) show an upregulation in the gene expression profile for the detached cells compared to the pellicle and the submerged. Classification by functional categories (Supplementary Fig. 4b) shows that 85 % of the motility and chemotaxis genes are in group GL9. This group contains genes of the *fla/che* operon and *hag*, the latter showing an upregulation by approximately 3Log2FC and 1.5Log2FC in the detached cells compared to the submerged and the pellicle, respectively. Interestingly, belonging to the *fla/che* operon in GL9, *swrD* encodes a swarming protein (SwrD) used to promote flagellar power. The *swrD* gene is the highest upregulated gene in the detached cells compared to the pellicle by a 2Log2FC (PL vs. DC). This could suggest that a rather high viscosity of the liquid culture requires a swarming-like process for efficient migration of the cells in the liquid column.

Example:

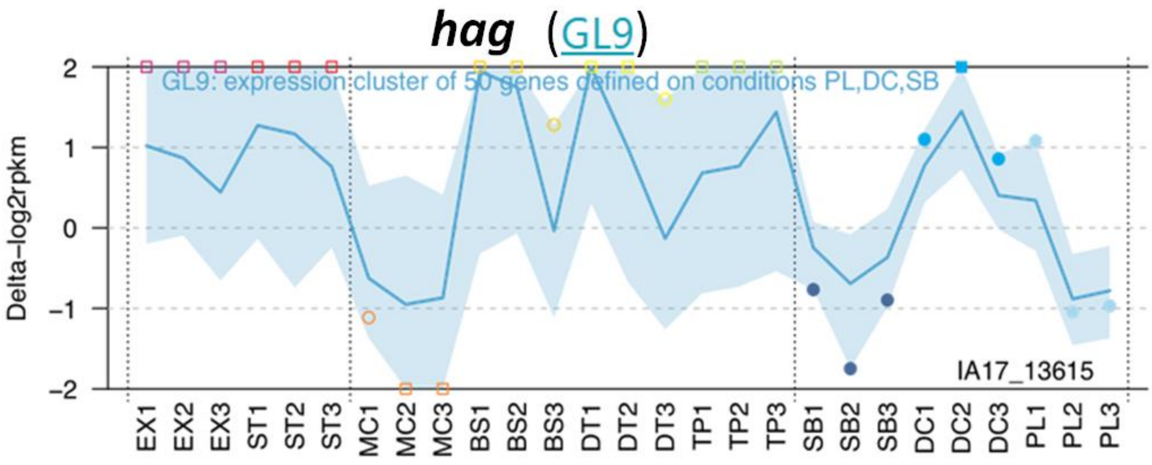

Expression profile across the different populations of the hag gene (flagellin protein essential for motility and chemotaxis). The graph was extracted from Genoscapist.

GL3 and GL4 groups contain most of the already known genes involved in biofilm formation (Fig. S6b), which are upregulated mainly in the pellicle (PL) and partially in the detached cells (DC) (Supplementary Fig. 6a). Biofilm genes in these groups are the *tapA* and *epsA-O* operons, *dhbACB*, *slrR*, *sinI*, *bslA*, and *spo0A*. Comparison between the matrix genes for the two biofilm populations after 24 hours for the liquid model indicates that the *tapA* operon is upregulated by 4Log2FC, the *epsA-O* operon by approximately 3Log2FC, and *bslA* by a Log2FC in the pellicle compared to the submerged (PL vs SB). As for the regulators encoding genes in this group,

slrR and *sinI* show an upregulation by 2LogFC and *spo0A* by a Log2FC in the pellicle compared to the submerged. Sporulation genes, of which about 87% are present in GL1 (Supplementary Fig. 6b), show a higher upregulation in the pellicle compared to the other two populations present, detached and submerged, like the *cge*, *cot*, *cwl*, *spoII*, *spoIII* , *spoIV*, *spoV* operons and genes. The most highly expressed genes in the pellicle compared to the submerged (PL vs. SB) by around 3Log2FC, are *ysxE*, *spoVID*, *spoIVA*, as those compared to the detached cells (PL vs. DC) are *cotC*, *yxeD*, *cotU*. The *ypqP* gene, potentially involved in the synthesis of polysaccharide, has been suggested to be involved in the addition of polysaccharides to the spore envelope. This gene is upregulated by 1.6Log2FC and 1.9Log2FC in the pellicle compared to the submerged or the detached cells, respectively.

Example:

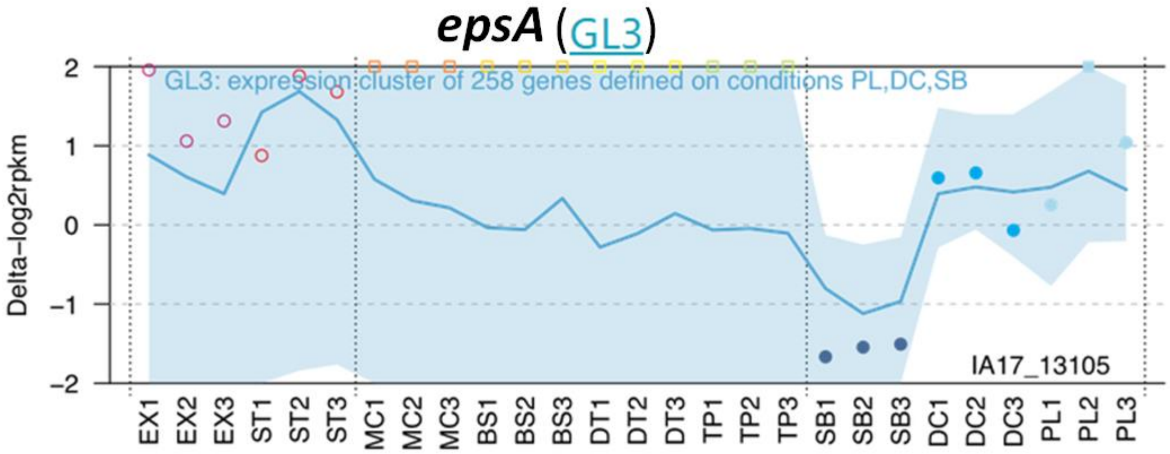

Expression profile across the different populations of the epsA-O operon (essential for matrix production). The graph was extracted from Genoscapist.

GL5, a group clustering 135 genes, is upregulated in the detached cells (DC) and submerged (SB) (Supplementary Fig. 6a). The *narG-I* operon, involved in nitrate respiration, is highly upregulated by a 5Log2FC in both detached and submerged cells compared to pellicle (PL vs. DC, and PL vs. SB). Carbon metabolism related genes, *i.e.*, *lctP*, *gapA*, *eno*, *cggR*, *ackA*, *pgm*… are all upregulated by more than Log2FC in the submerged compartments (SB and DC) compared to the pellicle (PL). Supplementary Fig. 6a, shows a high expression in the submerged population compared to the pellicle and the detached one in the GL2 clustering group. This group contains around 70% of genes related to the functional category coping with stress (Supplementary Fig. 6b), most of which are regulated by the SigB regulon *i.e.*, *yjgD*, *ygxB*, *csbx*, *ydbD*, *ktaE*, *yhxD*,... that are upregulated by 3Log2FC and 2Log2FC in the submerged compared to the pellicle or to the detached cells, respectively (downregulated in the comparisons PL vs. SB and DC vs. SB).

Example:

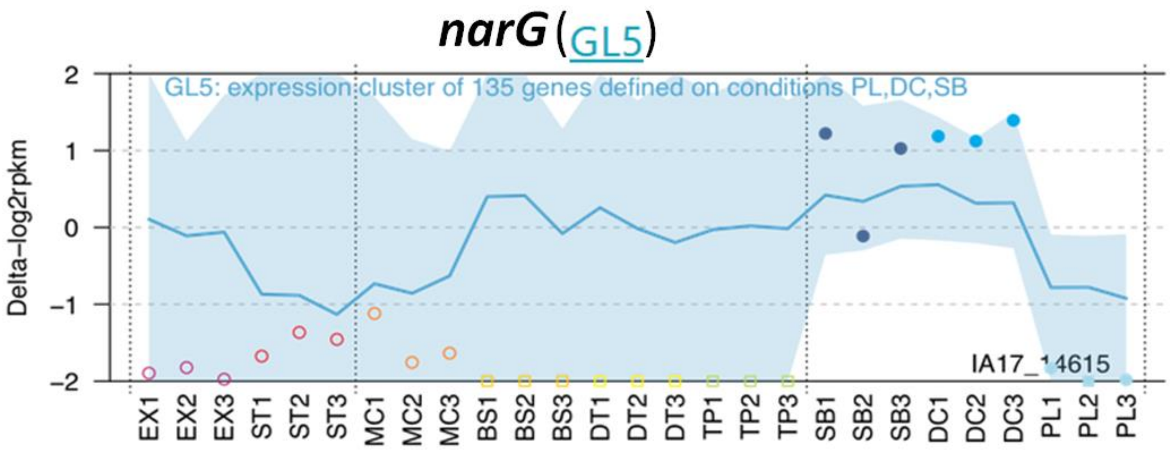

Expression profile across the different populations of the narG-I operon (anaerobic nitrate respiration). The graph was extracted from Genoscapist.

About 34 % of the differentially expressed genes between the different populations of the liquid model are poorly characterised or of unknown function (Supplementary Fig. 6b). Thus, in the comparison between the pellicle and the submerged (PL vs. SB), among others genes like *yoaC*, *yitJ*, and *ywcI* are upregulated while *yjgC*, *ydaC*, and *yxlE* are downregulated by 3Log2FC. Moreover, genes such as *ydjJ*, *yoxB*, and *yycD* are downregulated by more than 2Log2FC in the detached cells compared to the submerged (DC vs. SB). The *ywmC* and *ypzD* genes are upregulated by approximately 3Log2FC and *yclD*, *ybfA*, *yhbD*, and others are downregulated by more than 2Log2FC in the pellicle compared to the detached cells (PL vs. DC).

3- Supplementary Table

Table 1: Forward (F) and reverse (R) primers used for the construction of plasmids with reporter fusions.

Fusion	Primers	Plasmid
PepsA-gfpmut3	F: CCGCGGGCTTTCCCAGCCCTTTAACCGATCATC<br>R: GTTCCTCCTTCCCACCTTCAGCCTTCCCGCG	pBSB2epsA
PypqP-gfpmut3	F: CCGCGGGCTTTCCCAGCTTGCCAAACTCATAAGAATG<br>R: GTTCCTCCTTCCCACCTCCAACTCTCGTTTCTCTAC	pBSB2ypqP
PctaA-gfpmut3	F: CCGCGGGCTTTCCCAGCGTAAGAAGAACGGTGTTTATATTGCC<br>R: GTTCCTCCTTCCCACCCATACTGCTGCAATTTTATATACGTTC	pBSB2ctaA
PnarG-mCherry	F: CCGCGGGCTTTCCCAGCGGCAGTGTCCGTTTTATGGACAC<br>R: GTTCCTCCTTCCCACCCGAGTCAGGTGATGCTAAGTTCAC	pBSB8narG
PskfA-mCherry	F: CCGCGGGCTTTCCCAGCGCTGCCCTGCATCTCGGTTGTG<br>R: GTTCCTCCTTCCCACCAATTTTTGCATAGAGTCTATTGACATAG	pBSB8skfA
PcomGA-gfpmut3	F: CCGCGGGCTTTCCCAGCTCCGATTACAGCTCTGGGTGCC<br>R: GTTCCTCCTTCCCACCCGCATATTGTAGAAAAAGAAGAAAAGG	pBSB2comGA
PaprE-mCherry	F: CCGCGGGCTTTCCCAGCCTGCTATCAAAATAACAGACTCGTG<br>R: GTTCCTCCTTCCCACCAATTCAGAGTAGACTTACTTAAAAGAC	pBSB8aprE
PcggR-mCherry	F: CCGCGGGCTTTCCCAGCCGCCTATACATTTTGGATCTTTGCGGTGAT TAACAT<br>R: GTTCCTCCTTCCCACCCTTTTTTGCTGGACATTATATGTCCCGCTA	pBSB8cggR
capE-mCherry	F: CCGCGGGCTTTCCCAGCCAAGAGTATGACAATGATCCAAATG<br>R: GTTCCTCCTTCCCACCTGAATTATTTATTGGCGTTTACCGG	pBSB8capE
PspoIIGA-mCherry	F: CCGCGGGCTTTCCCAGCCGTTTACCATTCGTATGCCGCTGA<br>R: GTTCCTCCTTCCCACCCTTGCCTCACGCTGTTCCCCTTC	pBSB8spoIIGA
PspoVC-mCherry	F: CCGCGGGCTTTCCCAGCGAAGTTCCGATTCATCTGACCGGAG<br>R: GTTCCTCCTTCCCACCTCACATAACTCCCGTCTTCATAAAC	pBSB8spoVC
PtapA-mCherry	F: CCGCGGGCTTTCCCAGCGGTCCTTCAAAAAATGGAGGACC<br>R: GTTCCTCCTTCCCACCACACTGTAACTTGATATGACAATCG	pBSB8tapA
