# Supplementary material for: Direct comparison of spatial transcriptional heterogeneity across diverse Bacillus subtilis biofilm communities
Source: Nat Commun. 2023 Nov 20;14:7546. doi: 10.1038/s41467-023-43386-w (PMC10661151; doi:10.1038/s41467-023-43386-w)
Supplement: Supplementary file 3 — Description of Additional Supplementary Files [file 41467_2023_43386_MOESM3_ESM.pdf]

Description of Additional Supplementary Files:

Supplementary Data 1: Summary by gene of RNASeq data including normalised expression level in each sample, differentially expressed genes between conditions, and expression clusters.

Supplementary Data 2: Summary of the gene set enrichment analysis comparing expression clusters and Subtiwiki annotations (regulons and functional categories).

Supplementary Movie 1: Visualising dendritic swarming patterns of Bacillus subtilis using fluorescent reporter genes. Strain GM3924 was cultured under the swarming conditions detailed in the materials and methods section. Transcriptional fluorescent fusions within this strain report the expression of both hag and tapA, visualised by Gfp (in green) and mCherry (in red), respectively. Fluorescence images for the swarming colony experiment were captured every 5 minutes using a custom-made imaging device installed within a microbiological incubator (Heratherm IGS 100, ThermoFisher Scientific). Excitation was achieved using an LED light source (pE-4000, Coolled, UK). Image acquisition was performed with a CMOS camera (Cellcam Centro, Cairn Research, UK) with a macro lens (Navitar MVL7000, Thorlabs, USA), through a dual-band emission filter (59010m, Chroma, USA). The entire setup was controlled using µManager (http://www.micro-manager.org). The resulting movie illustrates the bacterial journey, starting from the point of inoculation at the centre of a 10 cm diameter plate. Over the initial 5 to 6 hours, bacterial proliferation coincides with the activation of the hag gene, a key contributor to motility. Around the 7-hour mark, a series of small bud-like structures emerge from the mother colony, gradually maturing into dendritic formations. As time advances, spanning approximately 14 to 15 hours, the bacteria within the mother colony begin expressing tapA, intricately involved in the assembly and cell wall anchoring of the TasA protein, essential for extracellular matrix synthesis. This intricate process culminates in the formation of a structured Mother Colony, an early biofilm Base, monolayer Dendrites and rapidly dividing motile cells at the Tips.

Supplementary Movie 2: Unveiling swarming behaviour: kinetic insights through brightfield microscopy. Using binocular microscopy (Axio Zoom V16, ZEISS) in relief contrast mode, the GM3924 strain was inoculated onto a 10 cm diameter plate. The movie perceptibly illustrates the sequential events during swarming. Within the initial hour, the production of the surfactin ring commences, progressively expanding across the plate as time advances. Concurrently, the bacteria at the point of inoculation undergo substantial proliferation, culminating in the formation of a microcolony at the site of inoculation. From this mother colony bacteria initiate outward growth in dendritic structures. Image acquisition was performed at 5-minute intervals, and subsequently three fields of view (each measuring 3.3x2.8mm) were stitched together to create a comprehensive representation.

Supplementary Movie 3: Unveiling swarming behaviour: kinetic insights through transcriptional fusions. Using binocular microscopy (Axio Zoom V16, ZEISS) in epifluorescence mode, the GM3924 strain, transcriptionally reporting the hag gene (by Gfp, in green) and tapA (by mCherry, in red), was

inoculated onto a 10 cm diameter plate. Image acquisition was performed at 5-minute intervals, and subsequently three fields of view (each measuring 3.3x2.8mm) were stitched together.

Supplementary Movie 4: Spatiotemporal observation of strain GM3900 reporting transcription of gapB by Gfp (in green) and of cggR by mCherry (in red) during submerged biofilm development. An image was taken every 90 minutes for 72 hours followed by Imaris 4D projection for display.

Supplementary Movie 5: Submerged biofilm dynamics of B. subtilis NDmed-GFP (GM3649) with propidium iodide. An image was taken every 1 hour for 48 hours at 30°C followed by Imaris 4D projection for display.

Supplementary Movie 6: Spatiotemporal observation of strain GM3924 reporting transcription of hag by Gfp (in green) and of tapA by mCherry (in red) during submerged biofilm development. An image was taken every 1 hour for 48 hours followed by Imaris 4D projection for display.

Supplementary Movie 7: Spatiotemporal monitoring of strain GM3346 swimmers during the submerged biofilm. A 30 seconds movie was taken every 1 hour for 48 hours at a position 10 µm away from the surface followed by Imaris 4D projection for display.

Supplementary Movie 8: Spatiotemporal observation of strain GM3903, reporting transcription of ackA by Gfp (in green) and of aprE by mCherry (in red) during submerged biofilm development. An image was taken every 1 hour for 48 hours followed by Imaris 4D projection for display.

Supplementary Movie 9: Spatiotemporal observation of strain GM3912 reporting transcription of comGA by Gfp (in green) and of skfA by mCherry (in red) during submerged biofilm development. An image was taken every 1 hour for 48 hours followed by Imaris 4D projection for display.

Supplementary Movie 10: Spatiotemporal monitoring of B. subtilis strains NCIB3610 (left movie) and NDmed (right movie) during submerged biofilm development. Bacterial cultures were maintained in synthetic B-medium and labelled with FM4-64 stain for visualisation. Images were captured at hourly intervals over a 48 hours period and subsequently processed using Imaris software for 4D projection and visualisation.
